# A novel immune-related gene signature for diagnosis and potential immunotherapy of microsatellite stable endometrial carcinoma

**DOI:** 10.1038/s41598-024-53338-z

**Published:** 2024-02-14

**Authors:** Yunyun Xiao, XiaoChuan Yu, Yaping Wang, Guangyao Song, Ming Liu, Daqing Wang, Huali Wang

**Affiliations:** 1 Department of Gynecology and Obstetrics, Dalian Maternal and Children’s Medical Group, No. 1 Dunhuang Street, Shahekou District, Dalian, 116033 Liaoning China; 2 Department of Pathology, Dalian Maternal and Children’s Medical Group, Dalian, 116033 Liaoning China; 3 Department of Oncology, Dalian Maternal and Children’s Medical Group, No. 1 Dunhuang Street, Shahekou District, Dalian, 116033 Liaoning China

**Keywords:** Computational biology and bioinformatics, Immunology

## Abstract

An immune-related gene signature (IRGS) was established to better understand the molecular and immunologic characteristics of microsatellite instable (MSI) and microsatellite stable (MSS) endometrial carcinoma (EC), and provide potential immunotherapy directions for MSS patients. Top 20 immune-related hub genes were screened by weight gene coexpression network analysis (WGCNA), and an IRGS was further established through Cox regression analysis. The molecular and immune characteristics were clarified in IRGS high and low risk groups. Expression and MS status validation of the IRGS were conducted through quantitative real-time Polymerase Chain Reaction (rt-qPCR) and immunohistochemistry (IHC) analysis. The IRGS includes 2 oncogenes (AGTR1 and HTR3C) and 2 tumor suppressor genes (CD3E and SERPIND1). Patients in IRGS high-risk group were more with MSS status, higher tumor grade, later FIGO stage, serous histology and elder ages compared with IRGS low-risk group (*P* < 0.05). Besides, patients in MSS group were more FIGO stages II–IV (42.7% vs. 26%), serous histology (35.7% vs. 5.3%) and with higher IRGS risk score (1.51 ± 3.11 vs. 1.02 ± 0.67) (*P* < 0.05) than patients in MSI group. Furthermore, patients in IRGS high-risk group had higher tumor purity, more Macrophages M1 and Macrophages M2 infiltrating, higher proportion of Macrophages M2 and Dendritic cells activated, lower proportion of T cells regulatory (Tregs), lower tumor mutation burden (TMB). Correspondingly, subjects in IRGS low-risk group had higher immunphenoscores than IRGS high-risk group. The relative mRNA level of AGTR1 and HTR3C were gradually increase, while CD3E and SERPIND1 were reversed in rt-qPCR. Through IHC experiments, AGTR1(69.2% vs 30%, P = 0.074) and HTR3C (76.9% vs 30%, P = 0.024) had higher positive staining rates in ECs than non-ECs. While SERPIND1 (84.6% vs 20%, P = 0.003) and CD3E (61.5% vs 40%, P = 0.000) had higher positive staining rates in non-ECs. IRGS is a potential diagnostic and prognostic biomarker for EC. IRGS low risk group might benefit from immune checkpoint inhibitors, while IRGS high risk group deserve other potential immunotherapy.

## Introduction

Endometrial cancer (EC) is the sixth malignant tumor among women in the world. According to the data on cancer of 185 countries announced by GLOBOCAN, there were about 417,367 new endometrial cancer patients in 2020, an increase of 9.2% over 2018, and about 97,370 new deaths in 2020^1,2^. The five-year survival rates of FIGO stages I and II patients ranged from 74 to 91%. While for FIGO stage III patients, the five-year survival rate was 57–66%. Worse still, the ratio was only 20–26% for FIGO stage IV patients, and 17% for extrapelvic recurrent patients^3^. Therefore, early detection and more effective treatments are urgently needed for metastatic and recurrent EC patients^4^.

The Cancer Genome Atlas (TCGA) endometrial project proposed four distinctive EC molecular subtypes according to genomic, transcriptomic and proteomic test results in 2013, including POLE-mutated (ultramutated) , microsatellite instability (hypermutated, MSI-H/mismatch repair deficiency, MMRd), copy number variation low (CNV-L) and copy number high (CNV-H) subtypes^5^. Approximately 30% of primary EC are MSI-H, and 13–30% of recurrent EC are MSI-H/MMRd. The National Comprehensive Cancer Network (NCCN) guidelines Version 1.2020 for EC included molecular subtyping as one of the prognostic risk factors, and immune checkpoint blockade (ICB) monotherapy or in combination with cytotoxic, other immunotherapy, target agents have been recommended as second-line treatment of EC^6–8^. The approved treatment pembrolizumab monotherapy, a Programmed cell death-1(PD-1) inhibitor, showed an objective response rate (ORR) of 53% and 57% for MSI-H advanced EC in two Phase II clinical trials. As for dostarlimab, also a PD-1 inhibitor, showed an ORR of 49% in MSI-H advanced EC versus an ORR of 20% in MSS tumors. Besides, the PD-L1 inhibitors avelumab and durvalumab have shown ORRs of 26.7% and 43% versus 6.25% and 3% in advanced dMMR tumors and MSS EC tumors respectively, as monotherapy^8–10^. Current major challenges include, who are candidates for anti PD-1/PD-L1 monotherapy, potential immune-resistence mechanisms emerging in MMRd EC and how to improve the immunogenicity of patients with MSS^11^.

To develop a new diagnostic, prognostic Immune-Related Gene Prognostic Signature (IRGS) as well as therapeutic targets for MSI and MSS EC, the transcriptome and clinical data of patients from the Cancer Genome Atlas database (TCGA) were analyzed through weighted gene coexpression network analysis (WGCNA) and prognosis regression analysis. Furthermore, the molecular and immunological characteristics of the gene signature were clarified, such as the connection with MS status, prognostic ability, correlation with the tumor microenvironment and immune infiltration. In addition, the experimental verification for the expression of the IRGS was also conducted, along with the MS status.

## Materials and methods

### Gene expression profile data

**Figure 1:**
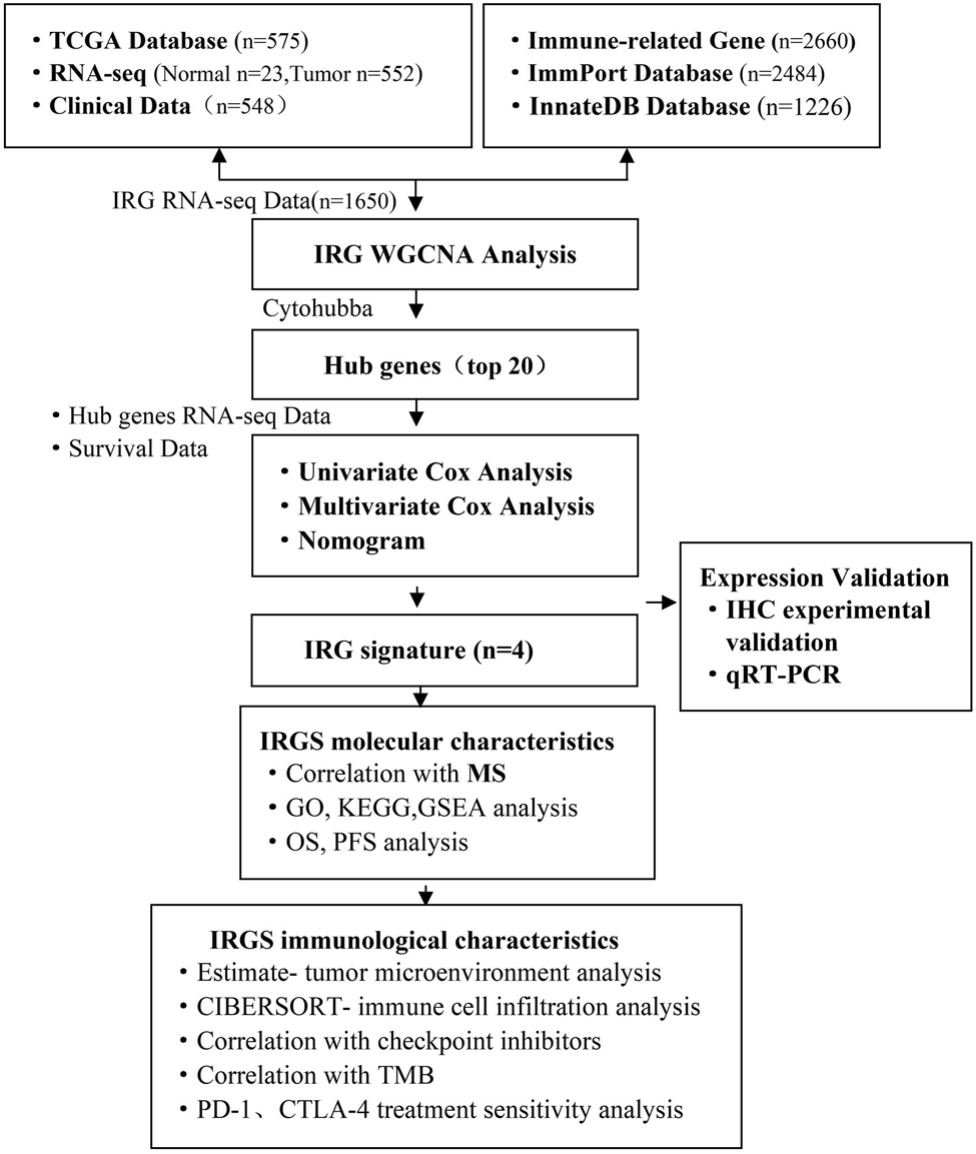
Workflow of the analysis process of this manuscript.

The RNA-seq data and clinical information of patients with EC were downloaded from the Cancer Genome Atlas database (TCGA, https://portal.gdc.cancer.gov/repository). Gene transcription data of 552 EC samples and 23 normal samples, clinical data of 544 EC patients were obtained. A total of 2484 immune-related genes were downloaded from the ImmPort database (https://www.immport.org/shared/home) and 1226 genes from the InnateDB database (https://www.innatedb.com/). After excluding duplicate genes, 2660 different immune-related genes were identified. The detailed workflow of this research is shown in Fig. 1.

### Hub IRGs identified through WGCNA

Weighted correlation network analysis (WGCNA) was conducted to identify hub immune-related genes based on WGCNA package in R (Version 4.3.1). First, the Pearson correlation coefficients between genes were calculated to obtain the similarity matrix. Then, the adjacency matrix of the genes was obtained by power calculation, amn =|cmn| β (amn is the adjacency coefficient between gene m and gene n, cmn is the Pearson correlation coefficient between gene m and gene n, β is the soft threshold value representing the correlation between genes). And the soft threshold value was tested by a scale-free graph. Finally, according to the WGCNA protocol, the adjacency matrix was converted into a topological overlap matrix to obtain a new distance matrix, gene modules (at least 5 genes per module) and a Tom (topological overlap matrix) diagram. The clustering distance between modules was set as 1-Tom, and a dynamic pruning tree was established to identify the module. Genes in the module were obtained and those with weight > 0.6 were shown in a network diagram. The network was drawn through Cytoscape (Version 3.9.1, www.cytoscape.org/), and “cytohubba” package was used to identify the top 20 hub genes.

### Prognostic analysis of the IRGS

Gene expression matrix of the top 20 hub genes was extracted from the TCGA RNA-seq data, and survival time corresponding to each patient was also gained. A univariate Cox proportional hazard regression analysis was employed to calculate the association between patient’s overall survival time and the gene expression level. Subsequently, a multivariate Cox proportional hazards regression analysis was used to screen out the immune-related gene signature (IRGS) to be independently prognostic factor for EC patients. The risk score of each patient was calculated as follows:

Risk score = β_1_* Gene_1_ Expression Level + β_2_*Gene_2_ Expression Level + … + βx* Gene_X_ Expression Level (β represents the regression coefficient). Based on the cut-off of the median risk score, patients were divided into high-risk and low-risk groups. Similarily, mutivariate Cox regression analysis including different clinical indexes and IRGS was conducted. The time-dependent receive operating characteristic (ROC) curves were drawn to check the regression analysis. Furthermore, a nomogram according to expression level of each IRG, clinical stage, tumor grade, MS status and age was drawn to predict patient’s survival outcome. And a calibration graph was used to evaluate the prediction ability. Besides, to verify the application value of IRGS, we separate the TCGA dataset into train data and test data through random number method. Clinicopathological features of each dataset were listed in Supplementary Table 6. The ROC and calibration curves were shown in Supplementary Fig. 5. All of the above analyses were performed in R, and *P*-value < 0.05 was set as the cut-off criterion for statistically significant.

### Molecular characteristics of the IRGS

Demographics of patients according to MS status from the TCGA database were analyzed. Furthermore, correlation of the IRGS with the clinical indexes, including MS status, age, clinical stage, tumor grade was calculated and exhibited in heatmaps. Survival and progression free analysis of IRGS were performed with Kaplan–Meier method (log-rank test, with a threshold of *P*-value < 0.05).

Gene set enrichment analysis (GSEA, version 4.1.0) was performed to predict the top 5 Kyoto Encyclopedia of Genes and Genomes (KEGG) pathways for each gene in IRGS with the clusterProfiler package. The differently expressed immune-related genes (DEIRGs) were explored through an edgeR package in R. An adjusted *P*-value (*AdjP*) < 0.05 and |log_2_FC|≥ 1 were considered as a DEIRG. Further, Gene ontology (GO) function enrichment and KEGG pathway analysis for the DEIRGs were conducted through the clusterProfiler package. Results were visualized as dot plots.

### Immunological characteristics of the IRGs

First, Estimation of Stromal and Immune cells in Malignant Tumour tissues using Expression data (Estimate) score of each patient was calculated to speculate the purity of the tumor cells in EC patients based on the estimate package. Second, the proportion of 22 types of infiltrating immune cells was obtained with CIBERSORT algorithm, and the summation of all estimate fractions equals 1 for each patient. The difference in tumor purity and immune cells percentage between the IRGS high and low expression groups were obtained with the limma package.

What’s more, genetic alterations information was obtained from the cBioPortal database to conduct the gene mutation analysis. Tumor mutation burden (TMB) between different IRGS groups was calculated subsequently. Forth, 47 immune checkpoint genes (ICGs) was identified through literature research. Correlation coefficients of each gene in IRGS with the ICGs were calculated. Finally, the immunophenoscore (IPS) of each patient to PD-1 and CTLA-4 was downloaded from the The Cancer Immunome Atlas (TCIA, https://tcia.at/home) database. Difference analysis of the IPS between two IRGS groups under anti-PD 1 or anti-CTLA 4 treatment was performed. Similarly, *P*-value < 0.05 was taken as statistically significant.

### Expression and MS status verification of the IRGS

Thirteen fresh specimens of endometrial carcinoma with different molecular types were collected in Dalian Women and Children’s Medical Group. The study was conducted in accordance with the Declaration of Helsinki. The research was approved by the ethics committee of Dalian Women and Children’s Medical Group and all patients were provided written informed consent. Inclusion Criteria: (1). Patients with primary endometrial carcinoma. (2). Patients accepted staging surgery. (3). Patients accepted molecular typing detection according to the Tans-PORTEC creteria. Exclusion Criteria: (1). Patients accepted chemotherapy or radiotherapy preoperation. (2). Patients with other serious systematic diseases. (3). Patients were pregnant or lactating. Patients’ clinicopathological information were also collected, including age, FIGO stage, tumor grade, pathologic patten, MS status, lymphatic vascular space infiltration status, muscular invasion status, lymph node status and so on. Besides, ten normal endometrium specimens were collected from patients with polyp during uterine curettage. Then these fresh specimens were embedded by paraffin.

### Quantative real-time PCR

Total RNA was extracted from collected tissues via Trizol reagent, and the concentration and purity of RNA was assessed using Micro Drop spectrophotometer. Then transcript RNA was reversed into complementary RNA (cDNA) with the PrimeScript RT reagent Kit (Takara, Dalian, China). Quantitative real-time PCR was conducted using TB Green Premix Ex Taq II (Tli RNaseH Plus) (Takara) on Heal Force CG-05. Glyceraldehyde 3-phosphate dehydrogenase (GAPDH) was used as endogenous control. Detailed primer sequences were listed as follows: AGTR1, Forward 5-ACCTGGCTATTGTTCACCCAAT-3’, Reverse 5-TGCAGGTGACTTTGGCTACAAG-3’; HTR3C, Forward 5-AGAAAGGCCTTCCGTCCATT-3’, Reverse 5-GGCAGACAGGGTGAAGGAGAT-3’; CD3E, Forward 5-AATTGGAGCAAAGTGGTTATTATGTCT-3’, Reverse 5-ACACACTCTTGCCCTCAGGTAGAG-3’; SERPIND1, Forward 5-AGTGGAGTCCCTGAAGTTGAT-3’, Reverse 5-TGAGATGCCTGCCATGTTG-3’. All experiments were repeated twice, and mRNA relative expression was calculated with 2−∆∆Ct method.                

### Immunohistochemistry

Furthermore, immunohistochemical experiments were conducted to verify the expression of the IRGS. Those Paraffin-embedded tissue sections (4 um thick) were de-paraffinized with xylene and dehydrated through graded ethanol. Then the sections were incubated with 3% hydrogen peroxide for 10 min to reduce the endogenous peroxidase activity. After adding sodium citrate buffer, the sections were placed in a microwave oven for 3 min twice for antigenic repair. Then sections were washed with PBS (phosphate-buffered saline) twice, and blocked with goat serum for 15 min. The slides were incubated with rabbit anti-AGTR1 primary antibody (Boster, BA0582) in a dilution of 1:200, rabbit anti-HTR3C antibody (Solarbio, K006945P) in a dilution of 1:50, rabbit anti-SERPIND1 antibody (Immunoway, YT2109) in a dilution of 1:200, and mouse anti-CD3E antibody (ZSGB-BIO, TA506064) in a dilution of 1:150 overnight at 4 °C separately. After washing, they were incubated for 30 min at 37 °C incubator with respective biotinylated goat anti-mouse/rabbit secondary antibody and biotinylated horseradish peroxidase complex. The sections were incubated with DAB (3,3′-diaminobenzidine tetrahydrochloride) for 5 min and counterstained with hematoxylin. After dehydration and sealing, the sections can be observed under microscopy.

The protein expression was evaluated by semi-quantitative method, both intensity and percentage of tumor cells staining were scored by 3 separate pathologists. The positive staining intensity range include: 0: no staining intensity; 1: weak stained (Faint Yellow); 2: moderate stained (claybank) and 3 were defined as strong stained (sepia), respectively. The proportion of positive tumor cells was scored subjectively as: 0 (0–5% staining), 1 (6–25% staining), 2 (26–50% staining), 3 (51–75% staining) and 4 (75–100% staining). The protein expression grade was evaluated by the product of the density score and the percentage score: scores ranged from 0 to 4 represent low grade, while scores that ranged from 5 to 12 were considered as high grade. And the total immunohistochemistry score was defined as the sum of the immunohistochemistry score of the IRGS (Oncogenic genes: positive expression recorded as 1 point, negative expression recorded as 0 point; Tumor suppressor genes: positive expression recorded as 0 point, negative expression recorded as 1 point). The differentially expressed levels of the IRGS between MSS and MSI EC patients from TCGA database were shown through Prism 8.0 (GraphPad, San Diego, CA, USA).

### Statistical analysis

Statistical analysis was conducted through R (Version 4.3.1) and Prism 8.0 (GraphPad, San Diego, CA, USA). Continuous quantitative data are presented as mean ± standard deviation (SD) and analyzed through Student t test. Categorical data are presented as numbers and percentages, and analyzed through Chi-square(X2) or Fisher exact test for non-ordinal variables, Mann-Whitney U test for ordinal variables. Mann–Whitney U test was used to test the difference of the total IHC socres between EC and non-EC groups, MSI and MSS groups. The positive rate of each gene in IRGS was analyzed with two-tailed analysis of Chi-square test and Fisher exact test. The differentially expressed levels of the IRGS between MSS and MSI EC patients from TCGA database were shown through Prism 8.0 (GraphPad, San Diego, CA, USA) using the Wilcoxon signed‐rank test, while the mRNA expression level were compared using the one-way analysis of variance or Kruskal-Wallis test. *P*-value was also two-sided and *P*-value < 0.05 was set as the cut-off criterion for statistically significant.

## Results

### Hub IRGs identified through WGCNA

**Figure 2:**
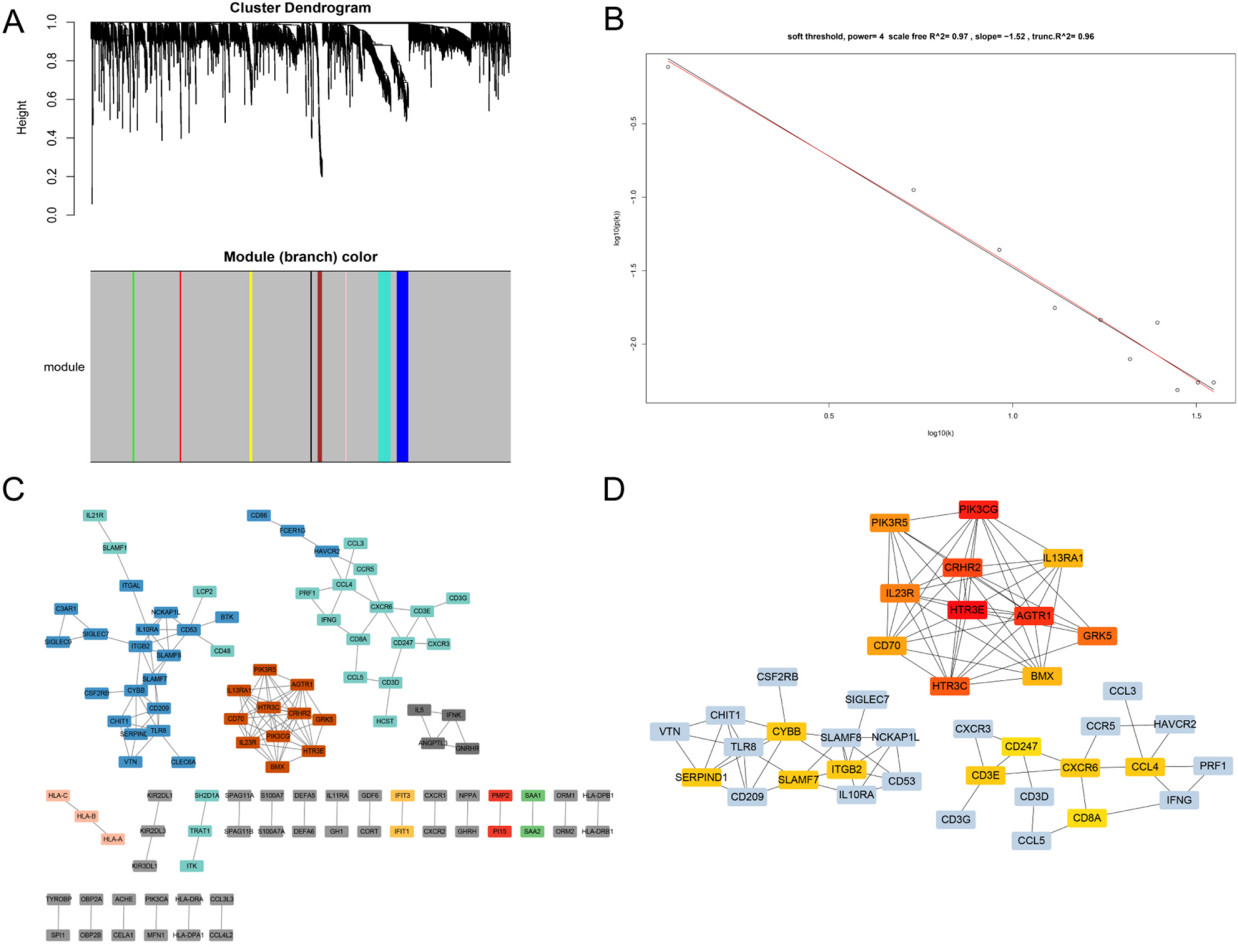
WGNCA analysis results of immune-related genes. (**A**) Immune-realted gene cluster dendrogram based on different metrics. (**B**) The scale free network of WGCNA analysis. (**C**) Netwrok of immune-related genes screened out through WGCNA analysis. (**D**) Subnetwork of top 20 hub immune-related genes.

Gene expression matrix of 1640 IRGs with gene expression normalization was finally obtained to conduct the WGCNA. The optimal soft-thresholding power was set as 4 (scale free R^2^ = 0.97) on the basis of the scale-free network. Nine modules were then discovered based on the average linkage hierarchical clustering (including the grey module, Fig. 2A), the scale free graph was shown in Fig. 2B. A total of 141 genes were allocated to another eight modules. Moreover, 100 genes were screened out with threshold weight > 0.6, and edges in the network were 139 (Fig. 2C). Then top 20 hub IRGs were obtained through cytohubba function (Fig. 2D). The relation matrix between modules and clinical traits of EC was shown in Fig. 2D.

### Prognostic regression analysis for screened IRGs

**Figure 3:**
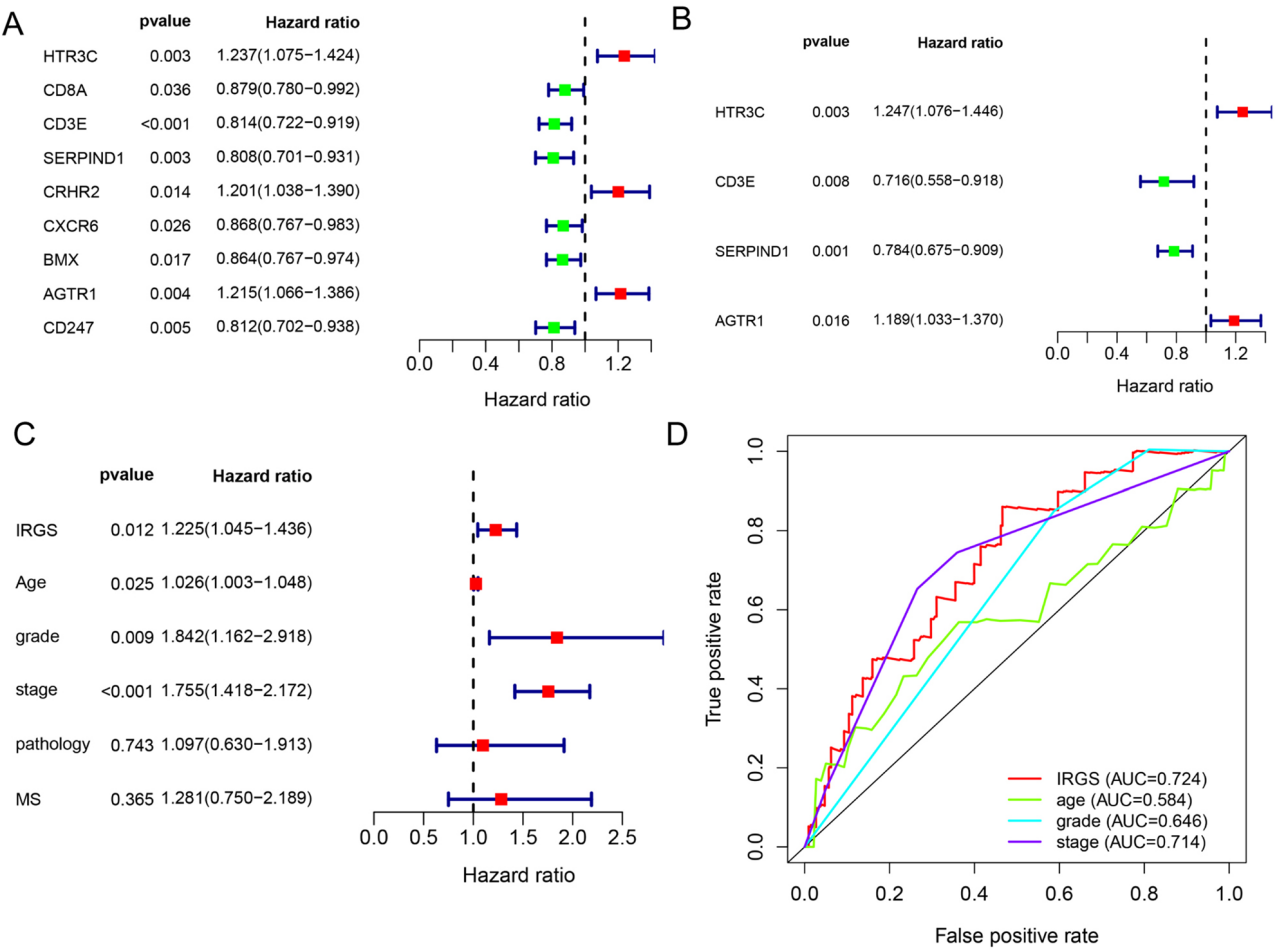
Prognostic regression analysis results of the screened immune-related genes. (**A**) Univariate Cox regression analysis results of the screened immune-related genes. (**B**) Multivariate Cox regression analysis results of the screened immune-related genes. (**C**) Multivariate Cox regression analysis results of the IRGS and other clinical indexes. (**D**) The receiver operating characteristic curves of IRGS and other three clinical indexes.

**Figure 4:**
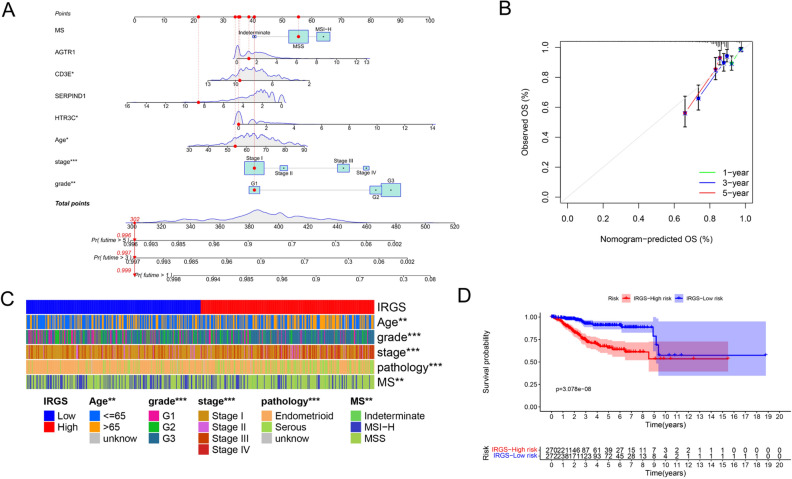
Molecular characteristics of the IRGS. (**A**) Nomogram based on the IRGS expression level and clinical characteristics to predict the survival of endometrial carcinoma patients. (**B**) The calibration graph examing the prediction ability of the nomogram. (**C**) Heatmaps of correlations among IRGS and different clinical characteristics. (**D**) Survival analysis results of the IRGS between different risk groups.

Through univariate Cox regression analysis, nine genes, including 3 oncogenes (HTR3C, CRHR2 and AGTR1), 6 tumor suppressor genes (CD8A, CD3E, SERPIND1, CXCR6, BMX and CD247) were proved to be correlated with the overall survival of EC patients (Fig. 3A). Subsequently, two carcinogenic genes (HTR3C, AGTR1), two tumor suppressor genes (SERPIND1, CD3E) were discovered to be independent prognostic factors of EC patients (Fig. 3B), which constituted the IRGS. Furthermore, the IRGS, age, pathologic grade and clinical stage were proved to be independently prognostic factors of EC (Fig. 3C). And according to Fig. 3D, the area under receiver operating characteristic curves (AUC) of IRGS was higher than other three clinical indexes, which was 0.724. Nomogram of the model based on the IRGS expression level and clinical characteristics to predict the survival of EC patients was shown in Fig. 4A. The calibration graph showed good prediction ability (Fig. 4B). What’s more, correlation of IRGS expression level with age, pathologic grade and types, clinical stage and MS status was displayed in Fig. 4C. Patients with higher IRGS expression are more with MSS, higher tumor grade, later FIGO stage, serous histology and elder ages (*P* < 0.05). Heatmaps of connections with different clinical characteristics for each IRG were shown in Supplementary Fig. 1. And the overall survival rates of patients in IRGS high-risk group were obviously lower than patients in IRGS low-risk group (Fig. 4D).

### Molecular characteristics of the IRGS

**Figure 5:**
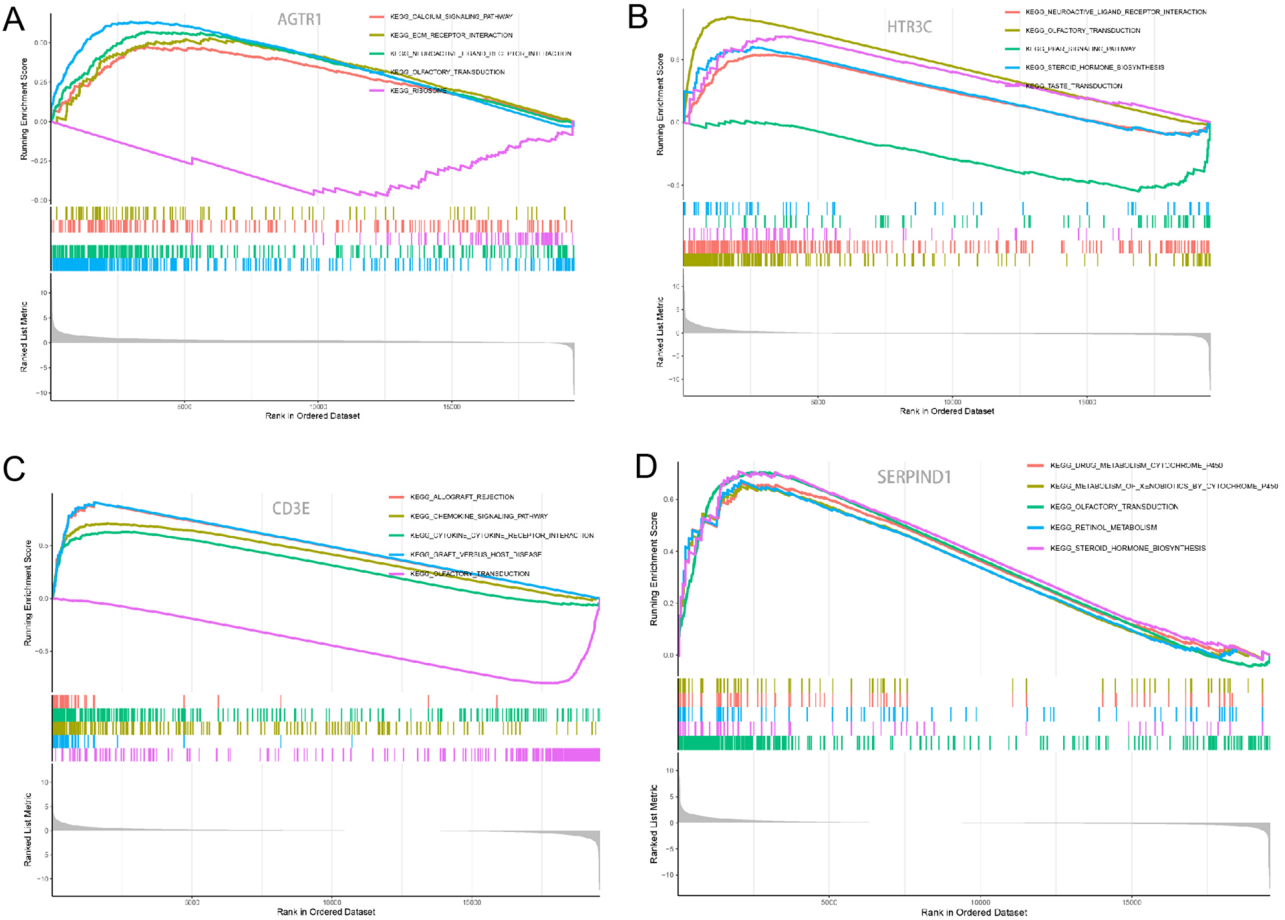
Gene set enrichment analysis results of IRGS.

Demographics of patients according to MS status from the TCGA database were shown in Table 1. Compared with patients in MSI-H group, more patients were FIGO stages II-IV (42.7% vs. 26%), more serous histology (35.7% vs. 5.3%) and with higher IRGS risk score (1.51 ± 3.11 vs. 1.02 ± 0.67) (*P* < 0.05) in MSS group. As shown in Fig. 5, the GSEA results show that most genes in IRGS were associated with olfactory transduction, steroid hormone biosynthesis, graft versus host disease. Pathway and functional enrichment analyses of GO and KEGG were conducted to search for the potential biological functions of DEIRGs. GO terms were divided into three parts: biological process (BP), cellular component (CC), and molecular function (MF). As a result, the top 5 enriched GO terms were exhibited in Supplementary Fig. 3A–C respectively. And the KEGG pathway results were shown in Supplementary Fig. 3D. Besides, HTR3C and CD3E were identified to have meaningful progression free survival curves (Supplementary Fig. 2B,C), but AGTR1 and SERPIND1 were not (Supplementary Fig. 2A,D).

**Table 1:** Demographics of patients from the TCGA database.

Characteristics	Samples (n = 530)	MS status		*P*
		MSS (n = 361)	MSI-H (n = 169)	
Age at operation				0.132
<= 65	301	197 (54.6%)	104 (56.8%)	
> 65	229	164 (45.4%)	65 (43.2%)	
FIGO stage				0.000*
I	332	207 (57.3%)	125 (74%)	
II	49	36 (10%)	13 (7.7%)	
III	120	94 (26%)	26 (15.3%)	
IV	29	24 (6.7%)	5 (3.0%)	
Grade				0.940
G1	98	68 (18.8%)	30 (17.8%)	
G2	118	78 (21.6%)	40 (23.7%)	
G3	314	215 (59.6%)	99 (58.6%)	
Pathology				0.000*
Endometrioid	392	232 (64.3%)	160 (94.7%)	
Serous	134	125 (34.6%)	9 (5.3%)	
Unknow	4	4 (1.1%)	0	
IRGS risk score		1.51 ± 3.11	1.02 ± 0.67	0.046*

MS: Microsatellite; MSI-H: Microsatellite instable; MSS: Microsatellite stable;FIGO: International Federation of Gynecology and Obstetrics; IRGS: Immune-related gene signature; *P < 0.05.

### Immunological characteristics of the IRGs

**Figure 6:**
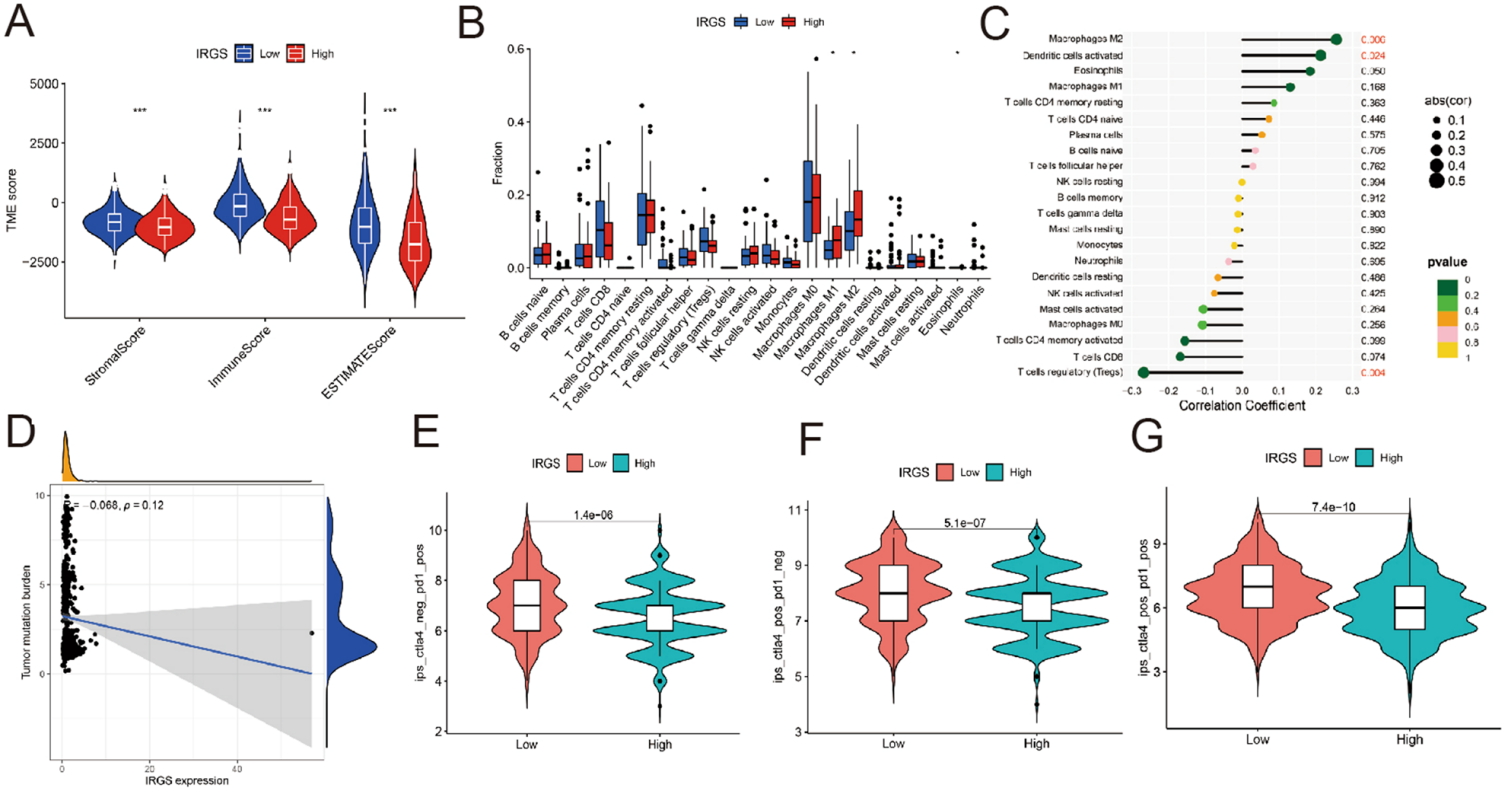
Immunological characteristics of the IRGS. (**A**) The Estimate analysis result of patients in different IRGS risk groups. (**B**) The immune infiltration analysis of patients between different IRGS risk groups according to the CIBERSORT algorithm. (**C**) Correlations of the expression level of IRGS with immune cells. (**D**) The tumor mutation burden (TMB) analysis between different IRGS risk groups. (**E**–**G**) Treatment sensitivity analysis to anti-PD 1 or anti-CTLA 4 therapy for patients with different IRGS risk.

Through Estimate analysis, patients in IRGS low-risk group had obviously higher Stromal Score, Immune Score and ESTIMATE Score, as shown in Fig. 6A. The result implied that patients in IRGS high-risk group had higher tumor purity than patients in IRGS low-risk group. Furthermore, according to the CIBERSORT algorithm, patients in IRGS high-risk group significantly had more Macrophages M1 and Macrophages M2 infiltrating than patients in IRGS low-risk group (Fig. 6B). Besides, the expression level of IRGS was positively correlated with the proportion of Macrophages M2 and Dendritic cells activated, while negatively correlated with the proportion of T cells regulatory (Tregs) (Fig. 6C). With the increase of IRGS expression, the tumor mutation burden (TMB) showed a relatively decreasing trend, but not statistically significant (R =  − 0.068, *P* = 0.12) (Fig. 6D). By evaluating the immunphenoscore of the patients under anti-PD 1 or anti-CTLA 4 treatment, subjects in IRGS low-risk group had higher scores than IRGS high-risk group, which represented high immunogenicity (Fig. 6E–G). Correlation of the four genes with common immune checkpoint genes (ICGs) was shown in Supplementary Fig. 4.

### Expression Validation of the IRGS

**Figure 7:**
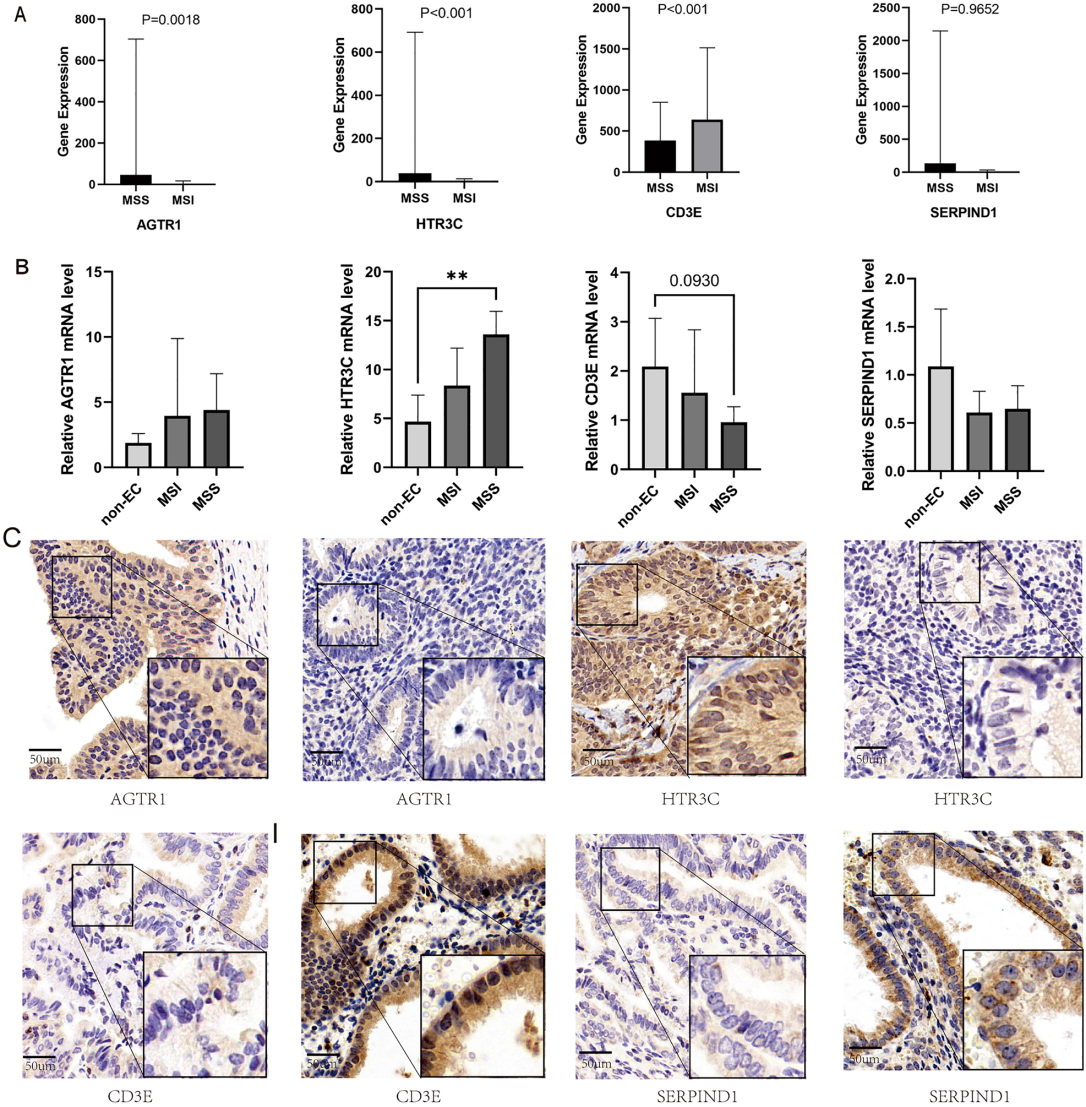
Expression verification of the IRGS. (**A**–**B**) Gene expression analysis of each gene in IRGS between Microsatellite stable (MSS), Microsatellite instable (MSI) and non-endometrial carcinoma status. (**C**) Immunohistochemistry results of each gene in IRGS (AGTR1, HTR3C, CD3E, SERPIND1) between endometrial carcinoma and normal endometrium tissues respectively.

The relative mRNA expression level of the IRGS were measured by qRT-PCR in 8 ECs (including 4 MSI ECs and 4 MSS ECs ) and 4 non-ECs. (as shown in Figure 7 B.) The expression level of AGTR1 and HTR3C gradually increase among non-EC, MSI EC and MSS EC tissues, while the expression of CD3E and SERPIND1 were reversed. The expression and location of the IRGS in EC were further assessed in 13 ECs and 10 non-ECs. And all of the EC patients had accepted molecular typing test. As Table 2 shown, AGTR1 (69.2% vs 30%, P = 0.074) and HTR3C (76.9% vs 30%, P = 0.024) had higher positive staining rates in ECs than non-ECs. While SERPIND1 (84.6% vs 20%, P = 0.003) and CD3E (61.5% vs 40%, P = 0.000) had higher positive staining rates in non-ECs. Further, the IHC scores for each EC patients were calculated, positive staining of AGTR1 or HTR3C recorded as 1 point separately, but negative staing of SERPIND1 or CD3E recorded as 1 point, otherwise were recorded as 0 point. Patients in EC group had obviously higher IHC scores than non-EC group, and with statistical significance (*P* = 0.001, Table 2). And patients in MSS group also had higher IHC scores (*P* = 0.036, Table 3). The differentially expressed levels of the IRGS between MSS and MSI EC patients from TCGA database, and the IHC staining results were shown in Fig. 7.

**Table 2:** The immunochemistry stain results of four novel screened IRGs.

IRG	Samples (n = 23)	IHC results		*P*
		Positive	Negative	
SERPIND1				0.003*
EC	13	2 (15.4%)	11 (84.6%)	
Non-EC	10	8 (80%)	2 (20%)	
AGTR1				0.074
EC	13	9 (69.2%)	4 (30.8%)	
Non-EC	10	3 (30%)	7 (70%)	
CD3E				0.000*
EC	13	5 (38.5%)	8 (61.5%)	
Non-EC	10	6 (60%)	4 (40%)	
HTR3C				0.024*
EC	13	10 (76.9%)	3 (23.1%)	
Non-EC	10	3 (30%)	7 (70%)	

*P < 0.05.

**Table 3:** The IHC score of the IRGS for patients from our medical center.

Groups	Samples (n = 23)	IHC scores					*P*
		4	3	2	1	0	
Tumor character							0.001*
EC	13	1	5	5	2	0	
Non-EC	10	0	0	1	4	5	
MS status							0.036*
MSI	5	0	0	4	1	0	
MSS	8	1	5	1	1	0	

EC: Endometrial carcinoma; Non-EC: Normal Endometrium; MS: Microsatellite; MSI: Microsatellite instable; MSS: Microsatellite stable; IHC: Immunohistochemistry; *P < 0.05.

## Discussion

The latest edition of FIGO Guidelines and NCCN Version 1.2023 for Gynecological Malignancies recommended immunotherapy and target therapy as the focus of research for EC^12^. With the wide application of TCGA molecular classification, ICB has been proved as an effective treatment, both as monotherapy and combination therapy. As mentioned above, what remain to be clarified include that who are candidates for anti PD-1/PD-L1 monotherapy, potential immune-resistence mechanisms emerging in MMRd EC and how to improve the immunogenicity of patients with MSS^11^. Therefore, it is necessary to better understand the molecular and immunologic characteristics of MSI and MSS EC patients.

Based on this, we analyzed the expression of immune-related genes in patients with endometrial carcinoma. First, top 20 hub genes were identified through WGCNA analysis, and these genes mainly distributed in 3 networks. Second, an immune-related gene prognostic signature were established via regression analysis, including 2 oncogenic genes (AGTR1, HTR3C) and 2 tumor suppressor genes (SERPIND1 and CD3E). Furthermore, the molecular and immunological characteristics as well as the protein expression conditions were clarified. In general, through bioinformatic analysis, the IRGS showed better ability to judge the prognosis of patients than clinical indicators, which could also distinguish different clinical features, especially the MS status. Besides, higher proportion of abnormal expression were identified via semi-quantitative protein analysis in patients from our medical center.

Angiotensin II type 1 receptor (AGTR1) is a high-affinity plasma membrane receptor of angiotensin II. Angiotensin II is a major peptipe effector of the renin-angiotensin (RAS) system, promoting hypertension, even proliferation and invasion of the tumor cells. Activation of AGTR1 could induce angiogenesis, cell proliferation, inflammatory responses, water and sodium retension^13^. Agnieszka et al. identified that angiotensin regulates KDR expression through AGTR1, thereby affecting local VEGF level in EC^14,15^. Several experiments identified that AGTR1 blockers (e.g. losartan) could act as an anticarcinogen. Boucher et al. identified that Losartan could down-regulate the expression of immunosuppressive and invasion-related genes in pancreatic ductal adenocarcinoma (PDAC), and this effect was associated with improved overall survival (OS). However, the combination of losartan with chemoradiotherapy (FOLFIRINOX + CRT) after chemotherapy can significantly reduce the number of immunosuppressive Treg and FOXP3 + tumor cells in PDAC lesions, and increase anti-tumor CD8 + T cell infiltration, so as to positively reshape the immunosuppressive microenvironment^16^. In this study, AGTR1 was proved to be higher expressed in MSS and later clinical stage patients. And in the IHC results, patients in EC group also had more positive expression, though not statistic significant (69.2% vs. 25%, *P* = 0.250). Therefore, AGTR1 blockers might play effective roles during immunotherapy for EC, especially for MSS patients.

The 5-hydroxytryptamine receptor 3C (HTR3C) is one of the 5-HT3 receptors family, which is activated by the 5-hydroxytryptamine (also called serotonin). It has been identified that serotonin could stimulate cancer cell growth in vitro, and is also associated with cancer cell migration, metastasis and angiogenesis^17,18^. Furthermore, 5HTRs antagonists, inhibitors of selective serotonin trasnporter (SERT) and serotonin synthesis have been applied to control cancer cell growth. In particular, the signal trasduction mechanism of 5-HT3 receptor is a ligand-gated ion channel (Na^+^/K^+^), the binding of serotonin resulting in plasma membrane depolarization, whereas other 5-HT receptors are G protein-coupled receptors (GPCRs) and activated by cAMP. And 5-HT3 receptor had been proved to be positively expressed in lung cancer and colorectal carcinoma^19,20^. Schneider et al. discovered that administration of antidepressants serotonin reuptake inhibitors (SSRIs) promoted the action of PD-1 checkpoint blockers and inhibited the tumor growth of untreatable pancreatic and colorectal cancers^21^. HTR3C was proved to be highly expressed in MSS patients in our study. Besides, patients in EC group also had higher positive expression rate than non-EC group (76.9% vs. 25%, P = 0.099) in the IHC results. So that serotonin inhibitors are potential combination of immunotherapy for MSS EC.

Serine proteinase inhibitors family D member 1 (SERPIND1), also called herparin cofactor II, can inhibit the function of thrombin through interaction with heparin^22,23^. Metelli et al. had identified that a direct link existed between thrombin catalytic activity and the release of mature transforming growth factor-β1 (TGF-β1) through cleaving the cell surface docking receptor glycoprotein A repetitions predominant (GARP) on platelet. And TGF-β1 had been proved to be associated with the cancer progression and immune evasion during the ICB immunotherapy. While systematic inhibition of thrombin could interrupt TGF-β1 releasate from platelet and improve antitumor immunity of the tumor mircroenvironment during immunotherapy^24^.Therefore, SERPIND1 might play anti-tumor role through inhibiting thrombin, and it is discovered to be under-expression in EC in this study. In the future, we would identify the correlation between SERPIND1, thrombin and TGF-β1. However, some researches had identified that SERPIND1 was upregulated in non small cell lung cancer (NSCLC), ovarian cancer and leukemia^25–27^.

CD3 antigen is an important marker on T cell surface, and non-covalent bond with TCR to form TCR/CD3 receptor complex. CD3 consists of four protein chains, namely CD3δ, CD3ε, CD3γ and CD3ζ, in which CD3δ/CD3ε, CD3γ/CD3ε compose the TCR-CD3 complex in the form of heterodimer with α/β chain of TCR. After antigen stimulation, the structure of the tail of CD3 cytoplasmic region underwent conformational changes. And downstream pathways, such as MAPK and NF-κB signaling pathways were activated, which can cause T cell proliferation, migration, cytokine production^28–30^. Current antibody drugs targeting CD3 usually recognize CD3E and activate T cells to kill the tumor cells. Up to now, more than 100 CD3-BsAb with different structures have been successfully developed and are mainly applied in hematological tumors. Application of CD3-BsAb in solid tumors remains to be investigated^31,32^. Considering that CD3E was obviously under-expression in EC than non-EC patients (61.5% vs. 0%, *P* = 0.082), especially in MSS patients, application of CD3-BsAb in the treatment of MSS EC deserves more research.

In addition, according to the immunological characteristics of IRGS, patients in IRGS high-risk group significantly had more Macrophages M1 and Macrophages M2 infiltrating than patients in IRGS low-risk group. Besides, the expression level of IRGS was positively correlated with the proportion of Macrophages M2 and Dendritic cells activated, while negatively correlated with the proportion of T cells regulatory (Tregs). And patients in IRGS low-risk group were more sensitive to ICB therapy. As patients in IRGS high-risk group were more MSS, we considered that tumor associated macrophages (TAM) might play a vital role in MSS patients. In the tumor microenvironment (TME), TAM could promote cancer progression and metastasis through promoting cancer cell proliferation and angiogenesis, inhibiting innate and adaptive immune responses^33^. Therefore, inhibition of the tumor-promoting roles of TAM is a potentially effective immunology treatment. Macrophage-based immunotherapy includes remodeling TAMs (Combining complement blockade, targeting interleukin-1, reprogramming TAM associated mRNA and MicroRNA), macrophages activator, targeting TAM metabolism and macrophage-based cellular immunotherapy (CAR-Macrophage)^34–38^. TAM had been proved to be associated with the development of the type I EC, but whether and how it contributed specifically in MSS EC still need more research^39^. According to the ESTRO/ESGO/ESP guidelines, TCGA molecular subtyping, FIGO stage, pathologic pattern, tumor grade, myometrial invasion and LVSI were acknowledged prognostic and therapeutic factors of endometrial carcinoma. At present, whether different molecular types of endometrial carcinoma with muscular infiltration deserve adjuvant therapy was still controversial, especially for P53 mutated patients^40^. Large retrospective cohort studies are necessary before conducting more prospective studies. In addition, with the application of multi-omics technology in cancer diagnosis and treatment^41,42^, it is necessary to combine metabolomics to study the application value of key metabolites in endometrial cancer.

## Conclusion

In conclusion, we identify a IRGS, a potential diagnostic and prognostic biomarker for EC. And IRGS high expression patients were more likely to be MSS status. IRGS low expression group might benefit from ICB therapy, while IRGS high expression group deserve other potential immunotherapy, including AGTR1 blockers (losartan), serotonin inhibitors, inhibition of thrombin, CD3-BsAb and Macrophage-based immunotherapy. And we will further explored these interventions in future studies.

### Supplementary Information

Supplementary Figure 1.

Supplementary Figure 2.

Supplementary Figure 3.

Supplementary Figure 4.

Supplementary Figure 5.

Supplementary Legends.

Supplementary Table 6.

Supplementary Tables.

## Data Availability

The TCGA and TCIA materials are public available (Detailed information can be obtained from the supplementary materials).
