# Supplementary figures and images for: A novel immune-related gene signature for diagnosis and potential immunotherapy of microsatellite stable endometrial carcinoma
Source: Sci Rep. 2024 Feb 14;14:3738. doi: 10.1038/s41598-024-53338-z (PMC10867009; doi:10.1038/s41598-024-53338-z)

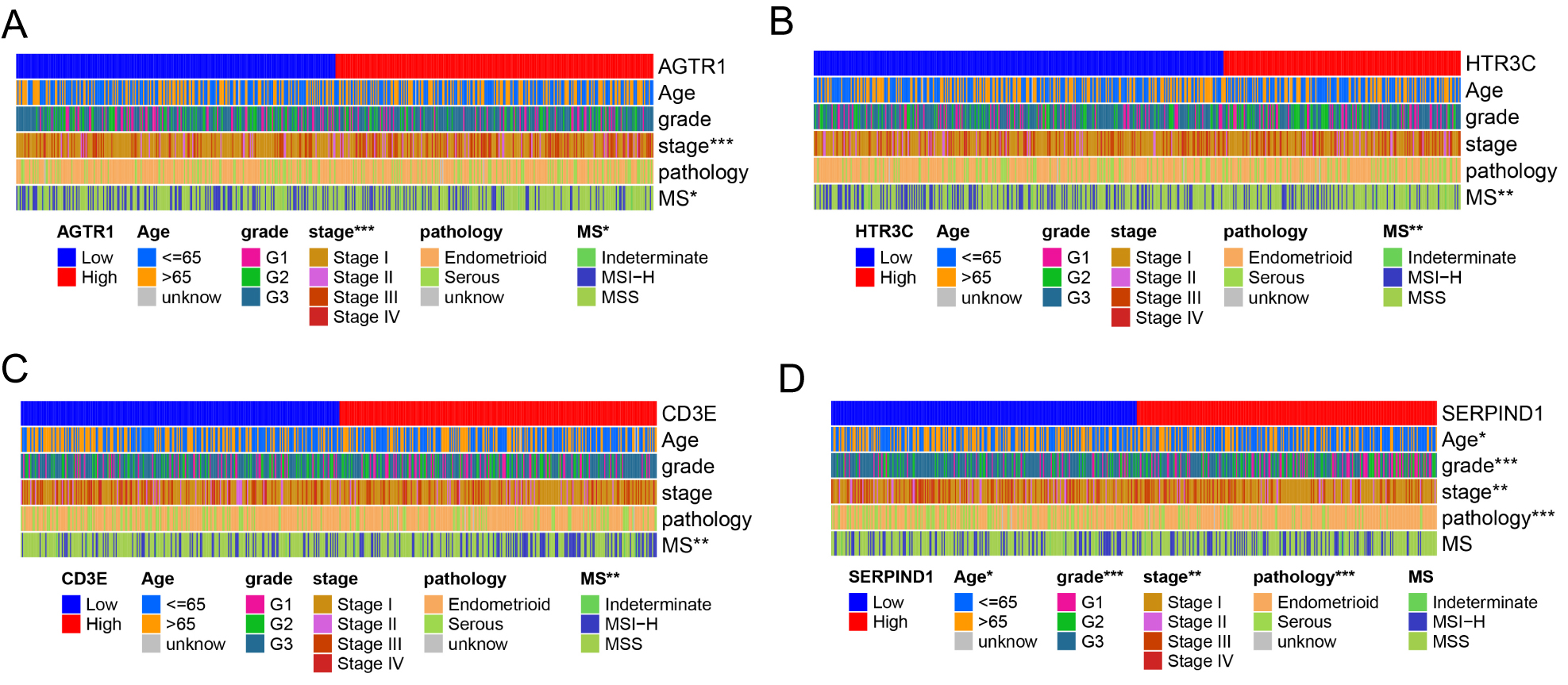

Supplement: Supplementary file 1 — Supplementary Figure 1. [file 41598_2024_53338_MOESM1_ESM.jpg]

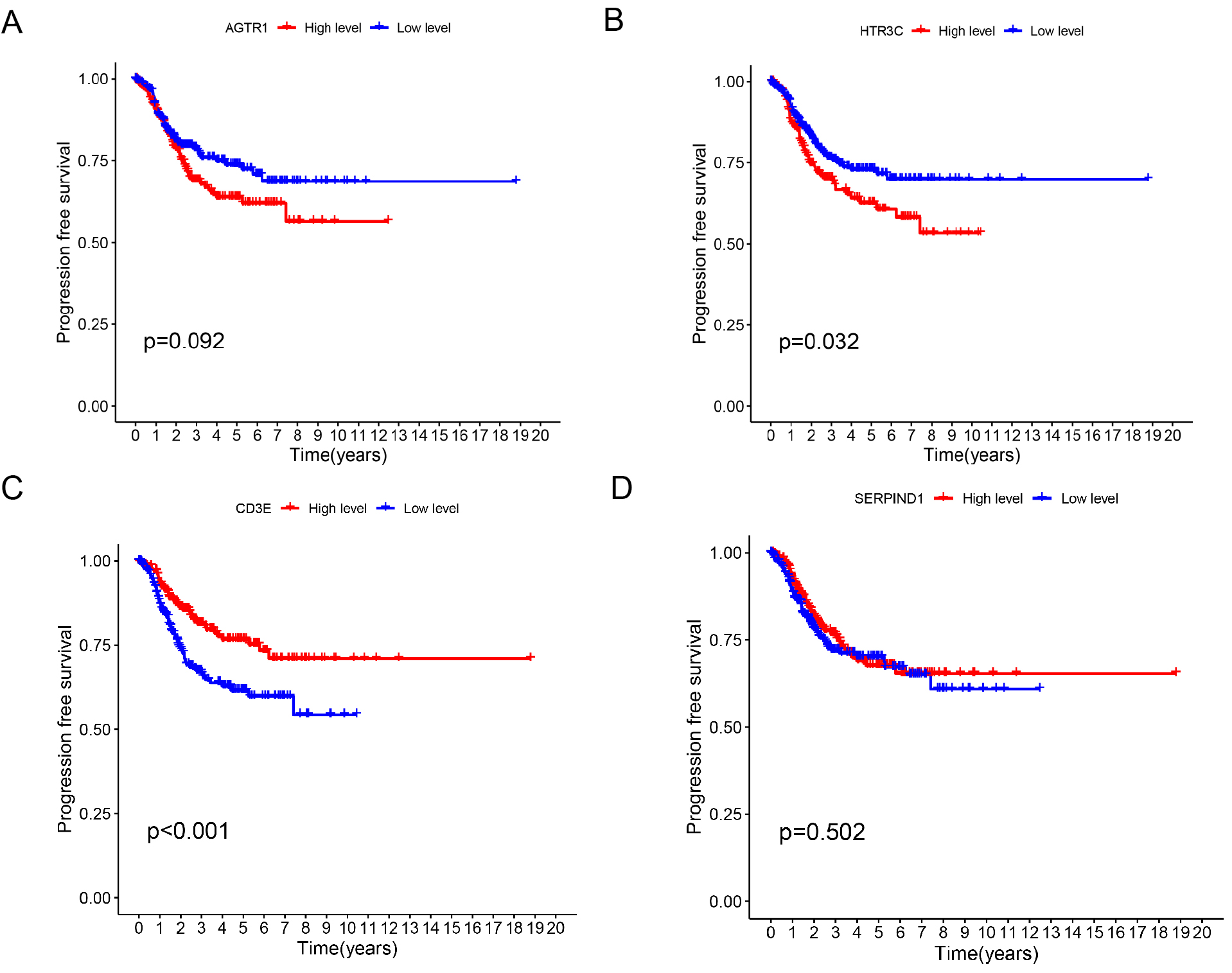

Supplement: Supplementary file 2 — Supplementary Figure 2. [file 41598_2024_53338_MOESM2_ESM.jpg]

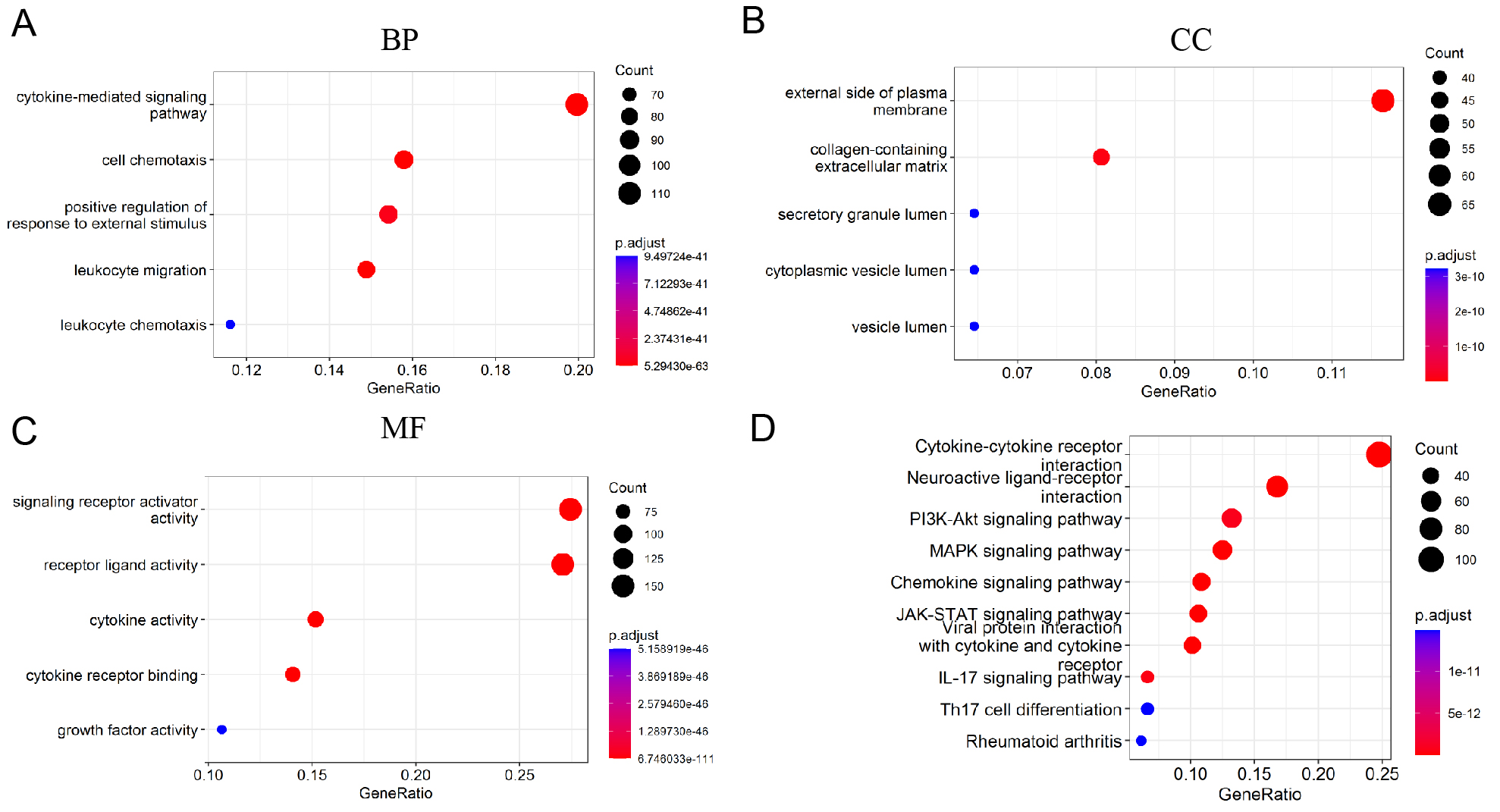

Supplement: Supplementary file 3 — Supplementary Figure 3. [file 41598_2024_53338_MOESM3_ESM.jpg]

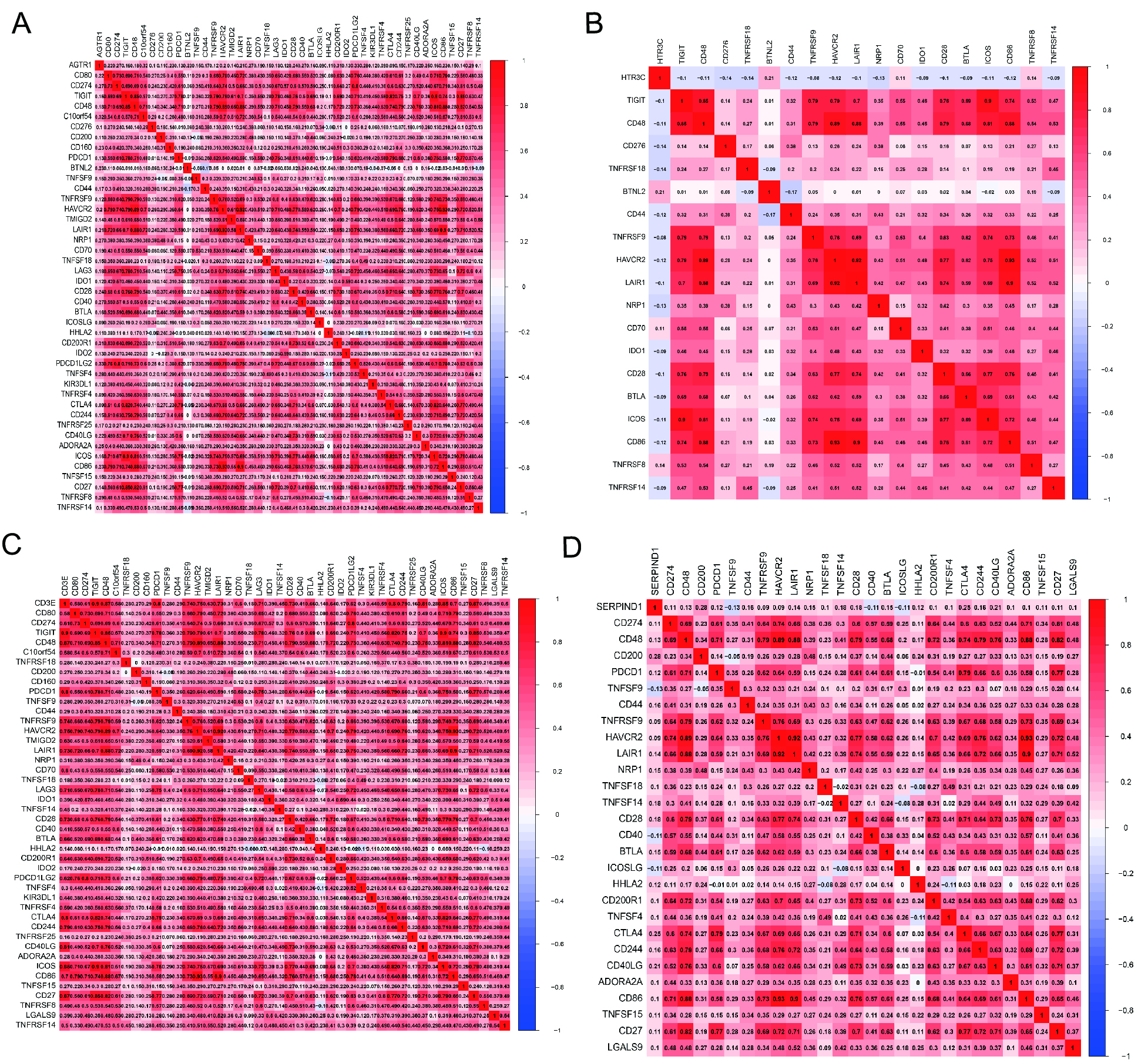

Supplement: Supplementary file 4 — Supplementary Figure 4. [file 41598_2024_53338_MOESM4_ESM.jpg]

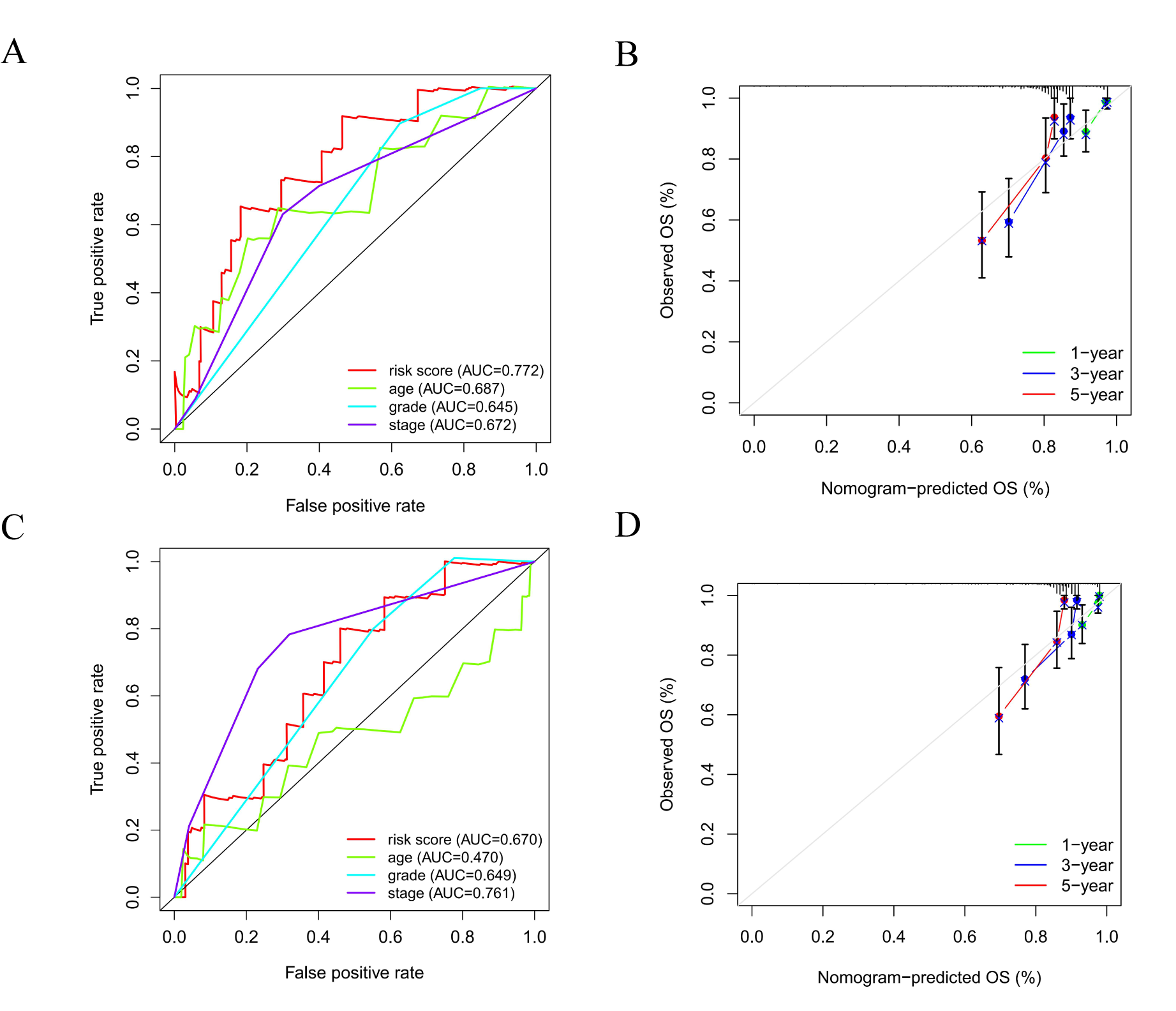

Supplement: Supplementary file 5 — Supplementary Figure 5. [file 41598_2024_53338_MOESM5_ESM.tif]
