# Supplementary material for: A novel immune-related gene signature for diagnosis and potential immunotherapy of microsatellite stable endometrial carcinoma
Source: Sci Rep. 2024 Feb 14;14:3738. doi: 10.1038/s41598-024-53338-z (PMC10867009; doi:10.1038/s41598-024-53338-z)
Supplement: Supplementary file 6 — Supplementary Legends. [file 41598_2024_53338_MOESM6_ESM.docx]

**Supplementary Figure and Table Legends**

**Supplementary Figure 1.**  Heatmaps of connections each gene in IRGS with different clinical characteristics.

**Supplementary Figure 2.** Progression free survival analysis results of each gene in IRGS.

**Supplementary Figure 3.** Gene ontology and KEGG pathway enrichment analysis for differentially expressed genes in endometrial carcinoma.

**Supplementary Figure 4.** Correlations of each gene in IRGS with immune checkpoint associated genes.

**Supplementary Figure 5.**  **(A)** The receiver operating characteristic curves of IRGS and other three clinical indexes in train data. **(B)** The calibration graph examing the prediction ability of the nomogram in train data. **(C)** The receiver operating characteristic curves of IRGS and other three clinical indexes in test data. **(D)** The calibration graph examing the prediction ability of the nomogram in test data.

**Supplementary Table 1.** The RNA-seq data downloaded from TCGA database.

**Supplementary Table 2.** A total of 2484 immune-related genes downloaded from the ImmPort database.

**Supplementary Table 3.** A total of 1226 genes obtained from the InnateDB database.

**Supplementary Table 4.** The immunophenoscore and other clinical information downloaded from the TCIA database.

**Supplementary Table 5.** The clinical information obtained from TCGA database.

**Supplementary Table 6.** Demographics of patients in train and test data from the TCGA database.
