# Supplementary material for: A novel immune-related gene signature for diagnosis and potential immunotherapy of microsatellite stable endometrial carcinoma
Source: Sci Rep. 2024 Feb 14;14:3738. doi: 10.1038/s41598-024-53338-z (PMC10867009; doi:10.1038/s41598-024-53338-z)
Supplement: Supplementary file 7 — Supplementary Table 6. [file 41598_2024_53338_MOESM7_ESM.docx]

Supplementary Table 6 Demographics of patients in train and test data from the TCGA database.

| **Characteristics** | **Train Data** | | | **Test Data** | | |
| --- | --- | --- | --- | --- | --- | --- |
|  | **MSS(n=179)** | **MSI-H(n=88)** | **P** | **MSS(n=182)** | **MSI-H(n=81)** | **P** |
| **Age at operation** |  |  | 0.227 |  |  | 0.370 |
| <=65 | 100(55.9%) | 56(63.6%) |  | 97(53.3%) | 48(59.3%) |  |
| >65 | 79(44.1%) | 32(36.4%) |  | 85(46.7%) | 33(40.7%) |  |
| **FIGO stage** |  |  | 0.001* |  |  | 0.029* |
| I | 93(52%) | 64(72.7%) |  | 114(62.6%) | 61(75.3%) |  |
| II | 21(11.7%) | 5(5.7%) |  | 15(8.2%) | 8(9.9%) |  |
| III | 51(28.5%) | 17(19.3%) |  | 43(23.6%) | 9(11.1%) |  |
| IV | 14(7.8%) | 2(2.3%) |  | 10(5.5%) | 3(3.7%) |  |
| **Grade** |  |  | 0.948 |  |  | 0.924 |
| G1 | 27(15.1%) | 13(14.8%) |  | 41(22.5%) | 17(21%) |  |
| G2 | 39(21.8%) | 20(22.7%) |  | 39(21.4%) | 20(24.7%) |  |
| G3 | 113(63.1%) | 55(62.5%) |  | 102(56%) | 44(54.3%) |  |
| **Pathology** |  |  | 0.000* |  |  | 0.000* |
| Endometrioid | 109(60.9%) | 83(94.3%) |  | 123(67.6%) | 77(95.1%) |  |
| Serous | 68(38%) | 5(5.7%) |  | 57(31.3%) | 4(4.9%) |  |
| Unknow | 2(1.1%) | 0 |  | 2(1.1%) | 0 |  |
| **IRGS** risk score | 1.70±4.25 | 1.02±0.71 | 0.023* | 1.32±1.15 | 1.02±0.64 | 0.031* |
